# Effect of a Standardized Family Participation Program in the ICU: A Multicenter Stepped-Wedge Cluster Randomized Controlled Trial*

**DOI:** 10.1097/CCM.0000000000006093

**Published:** 2023-11-07

**Authors:** Boukje M. Dijkstra, Paul J.T. Rood, Steven Teerenstra, Anne M.F. Rutten, Crista Leerentveld, Dominique C. Burgers-Bonthuis, Barbara Festen-Spanjer, Toine Klarenbeek, Mark Van Den Boogaard, Esther Ewalds, Lisette Schoonhoven, Johannes G. Van Der Hoeven, Lilian C.M. Vloet

**Affiliations:** 1 Research Department Emergency and Critical Care, School of Health Studies Nijmegen, HAN University of Applied Sciences, Nijmegen, The Netherlands.; 2 Department of Intensive Care Medicine, Radboud University Medical Center, Nijmegen, The Netherlands.; 3 Department for Health Evidence, Section Biostatistics, Radboud University Medical Center, Nijmegen, The Netherlands.; 4 Department of Intensive Care Medicine, Elisabeth Tweesteden Hospital, Tilburg, The Netherlands.; 5 Department of Intensive Care Medicine, ISALA Hospital, Zwolle, The Netherlands.; 6 Department of Intensive Care Medicine, Hospital Rijnstate, Arnhem, The Netherlands.; 7 Department of Intensive Care Medicine, Hospital Gelderse Vallei, Ede, The Netherlands.; 8 Department of Intensive Care Medicine, Máxima Medical Center, Veldhoven, The Netherlands.; 9 Department of Intensive Care Medicine, Bernhoven, Uden, The Netherlands.; 10 Nursing Science, Julius Center for Health Sciences and Primary Care, University Medical Center Utrecht, Utrecht University, Utrecht, The Netherlands.; 11 School of Health Sciences, Faculty of Environmental and Life Sciences, University of Southampton, Southampton, United Kingdom.; 12 IQ Healthcare, Radboud Institute for Health Sciences, Radboud University Medical Center, Nijmegen, The Netherlands.; 13 Foundation for Family and Patient Centered Intensive Care, Alkmaar, The Netherlands.

**Keywords:** anxiety, critical care, depression, essential care, family-centered care, family participation, intensive care unit, nursing, post-intensive care syndrome, posttraumatic stress disorder, relatives, satisfaction

## Abstract

**OBJECTIVES:**

To determine the effect of a standardized program for family participation in essential care activities in the ICU on symptoms of anxiety, depression, posttraumatic stress and satisfaction among relatives, and perceptions and experiences of ICU healthcare providers (HCPs).

**DESIGN:**

Multicenter stepped-wedge cluster randomized controlled trial.

**SETTING:**

Seven adult ICUs, one university, and six general teaching hospitals.

**PARTICIPANTS:**

Three hundred six relatives and 235 ICU HCPs.

**INTERVENTIONS:**

A standardized program to facilitate family participation inpatient communication, amusement/distraction, comfort, personal care, breathing, mobilization, and nutrition.

**MEASUREMENTS AND MAIN RESULTS:**

Data were collected through surveys among relatives and ICU HCPs. There were no significant differences in symptoms of anxiety in relatives in the intervention period compared with the control period (median Hospital Anxiety and Depression Scale [HADS] 5 [interquartile range (IQR) 2–10] vs 6 [IQR 3–9]; median ratio [MR] 0.72; 95% CI, 0.46–1.13; *p* = 0.15), depression (median HADS 4 [IQR 2–6] vs 3 [IQR 1–6]; MR 0.85; 95% CI, 0.55–1.32; *p* = 0.47) or posttraumatic stress (median Impact of Event Scale-Revised score 0.45 [IQR 0.27–0.82] vs 0.41 [IQR 0.14–1]; MR 0.94; 95% CI, 0.78–1.14; *p* = 0.54). Reported satisfaction was slightly lower in the intervention period (mean 8.90 [sd 1.10] vs mean 9.06 [sd 1.10], difference –0.60; 95% CI, –1.07 to –0.12; *p* = 0.01). ICU HCPs perceived that more relatives knew how to participate: 47% in the intervention period versus 22% in the control period (odds ratio [OR] 3.15; 95% CI, 1.64–6.05; *p* < 0.01). They also reported relatives having sufficient knowledge (41% vs 16%; OR 3.56; 95% CI, 1.75–7.25; *p* < 0.01) and skills (44% vs 25%; OR 2.38; 95% CI, 1.22–4.63; *p* = 0.01) to apply family participation.

**CONCLUSIONS:**

Application of a standardized program to facilitate family participation did not change mental health symptoms in relatives of ICU patients 3 months after discharge. ICU HCPs reported increased clarity, knowledge, and skills among relatives and ICU HCPs.

**KEY POINTS**

**Question:** What are the effects of a standardized program for family participation in essential care activities in the ICU?

**Findings:** This multicenter stepped-wedge cluster randomized controlled trial found that application of a family participation program led to increased clarity, knowledge, and skills regarding family participation in essential care activities among relatives and ICU healthcare providers, but did not lead to a relevant change in post-intensive care syndrome-family symptoms nor satisfaction levels.

**Meanings:** Use of a family participation program improves and facilitates family participation, but does not change post-intensive care syndrome-family symptoms nor satisfaction levels in relatives at three months post ICU discharge.

ICU stay is commonly experienced as stressful by patients and relatives. Also, ICU survivors report long-term physical, cognitive, and/or mental impairments, also known as “post-intensive care syndrome” (PICS) (1). Relatives may experience mental health symptoms, defined as PICS-family (PICS-F), possibly impacting their life for years (2).

Enabling relatives to participate in essential care activities may decrease relatives’ stress and anxiety and prepare them for their role as informal caregivers after ICU discharge. Though most relatives are willing to participate in essential care, currently a minority participates, suggesting that additional efforts from ICU healthcare providers (HCPs) may be necessary (3). Although most HCPs are willing to facilitate family participation, currently, practical guidance is lacking, and the fundamental evidence base for the benefits and limitations of family participation in adult ICU patient care is scarce (4, 5).

Recently, the application of a standardized program to facilitate family participation in daily ICU practice was pilot-tested in three centers. Both HCPs and relatives considered the use of such a program feasible and applicable. Important conditions were individual tailoring and provision of sufficient time for HCPs (6). However, the effects on patients’, relatives’, and HCPs’ outcomes and experiences remain unclear.

Therefore, the aim of this study was to determine the effect of a standardized family participation program in the ICU on satisfaction and mental health of relatives. We also studied HCP perceptions and experiences related to the program.

## MATERIALS AND METHODS

### Design

The “EFfect of FAMily PARTicipation” in essential care (EFFAMPART) study was a prospective multicenter stepped-wedge cluster randomized controlled trial, including patients, their relatives, and HCPs. In our study centers (clusters) were randomized, and crossed over every consecutive month from control to intervention (7). The study was conducted from May, 2021, to October, 2022. ICUs of seven centers (one university and six general teaching hospitals; clusters) in The Netherlands participated. The Medical Research Ethics Committee (MREC) Arnhem-Nijmegen waived the need for informed consent (MREC number 2020/7002).

### Participants

We included relatives of adult ICU patients (admission > 24 hr) where family participation was considered feasible according to local working group members. Participants were informed via information posters in the patient and family rooms and received a letter containing information about the study after recruitment. Relatives were excluded when patients received palliative care, since grief may influence outcomes such as symptoms of anxiety, depression, posttraumatic stress, and satisfaction. Patients or relatives who objected to study participation, relatives were unable to participate, or were not willing or able to complete the questionnaires were also excluded.

HCPs who were involved in the application of family participation were included in the survey.

### Interventions

**Figure 1.**
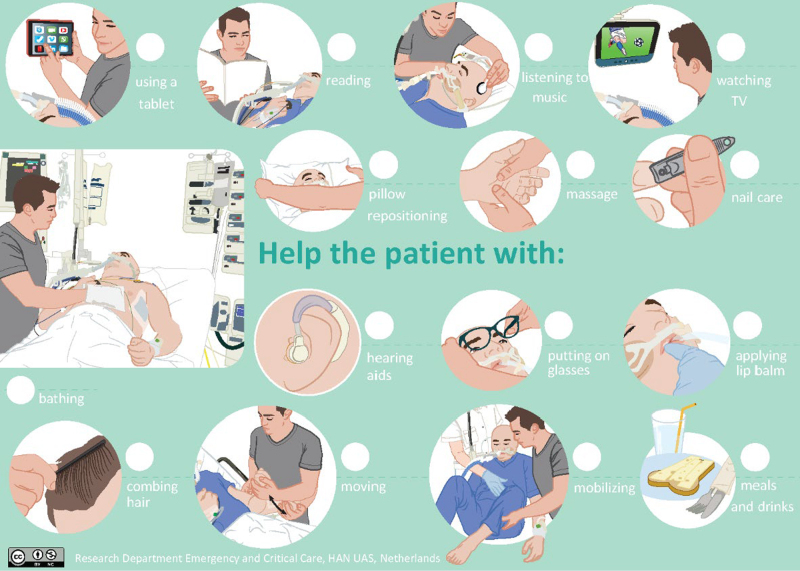
The Effect of Family Participation program menu. The menu below was adapted for low-literacy relatives or relatives from different cultural backgrounds, who have difficulty reading Dutch, printed double-sided and laminated. It contains similar information with pictures on the front and text on the back (Supplemental file 1, http://links.lww.com/CCM/H442).

A previously developed and pilot-tested standardized program to facilitate family participation in essential care activities in the ICU (8–10), was used in daily ICU practice (6). The EFFAMPART program contained 34 general activities such as assisting in communication, providing amusement and/or distracting activities, facilitating comfort, activities of daily living (shaving, combing hair, bathing), assisting with breathing exercises, mobilization, and nutrition (**Fig. 1**; **Supplemental files 1** and **3**, http://links.lww.com/CCM/H442).

At study onset, all ICUs started in the control period. Family participation was already informally applied, albeit not structurally facilitated nor documented in local protocols.

The implementation of the EFFAMPART program consisted of four steps. First, a local working group was formed in each ICU. This group guided the implementation in the individual ICU and consisted of a mix of ICU nurses, family care nurses, physicians, and research nurses and/or secretarial staff, complemented by one of the researchers (B.D., P.R.). The working group tailored the implementation strategy to the individual ICU and approved the template protocol and study materials prefabricated by the researchers (**Supplemental file 2**, http://links.lww.com/CCM/H442). Cookie jars with the study logo and logos of participating centers were distributed and filled monthly as a daily reminder.

Second, according to the stepped-wedge design, after a control period, ICU nurses were trained for 1 month. After the training period, family participation was considered standard care. Live and digital training sessions were provided, by the researchers (B.D., P.R.), aiming at training at least 70% of the ICU nursing staff. The sessions contained information about the current scientific knowledge of PICS(-F), anticipated possible beneficial effects of family participation, instructions on how to apply the EFFAMPART program, and statements from relatives that had experienced the added value of family participation.

Third, additional information about the study was provided with local online publication of the protocol, laminated posters for both relatives and HCPs (Supplemental file 2, http://links.lww.com/CCM/H442) and information and invitation letters for eligible relatives, also addressing safety precautions and the voluntary nature.

Fourth, the local working group frequently updated the ICU team about the study progress, based on feedback and newsletters from the primary researchers (B.D., P.R., L.V.).

### Outcomes

The primary outcomes were symptoms of anxiety, depression, posttraumatic stress, and satisfaction in relatives.

Symptoms of anxiety and depression were assessed 3 months after the patient’s discharge, using the Hospital Anxiety and Depression Scale (HADS), a validated instrument to assess symptoms of anxiety (HADS-A) and depression (HADS-D) (11). Both HADS-A and HADS-D consist of seven items, scored on a 4-point Likert scale (0–3), of which a sum score (0–21) is derived. A sum score of greater than or equal to 8 indicates significant symptoms of anxiety or depression (12, 13). Symptoms of posttraumatic stress disorder (PTSD) were assessed with the Impact of Event Scale-Revised (IES-R). This validated instrument consists of 22 items, scored on a 5-point Likert scale (0–4) (14). Afterward, a mean total score is calculated, with a mean score of greater than or equal to 1.6 indicating symptoms of PTSD (15).

Satisfaction of relatives was assessed at the patient’s discharge from ICU, using the “Consumer Quality Index for Relatives in the ICU” (CQI R-ICU) (11), consisting of 58 items that provide quality information about the experiences of relatives of ICU patients and assesses the dimensions “communication” and “participation.” Because the CQI R-ICU does not aggregate in a total score, a pragmatic numeric rating score (0 = very unsatisfied–10 = very satisfied) for satisfaction was added.

Secondary outcomes were differences in reported perceptions and experiences of HCPs. Therefore, a survey was sent to the HCP teams twice, at the end of the control period and the end of the study. The survey contained questions and statements on perceptions, changes in daily care provision, and contextual factors, related to implementation of the program, and investigated the proportions of HCPs that had a neutral to positive attitude toward family participation in essential care. Furthermore, the patient’s length of ICU stay, number of days on mechanical ventilation as well as incidents and adverse effects were monitored.

Intervention delivery was assessed with comparison of the reported participation rates, according to the CQI R-ICU. During the intervention period, the number of applied essential care activities was investigated.

### Sample Size, Randomization, and Blinding

The study was powered on 6 clusters and 10 measurement periods. Each participating center was considered a cluster and measurement periods were 1 month. As PICS-F scores do not aggregate into a total score, satisfaction levels were used for power calculation. A difference between the intervention group and the control group of 5% or more increase in satisfaction was considered clinically relevant. We estimated to provide more than 80% power to detect a difference with an α = 0.05 and intracluster correlation coefficient = 0.01, thus accounting for possible loss of patients and/or one ICU, requiring approximately 240 patients (16). The start of the intervention at center level was randomized, blinding was not possible. Because the study was considered low-risk, no interim analyses were executed.

### Statistical Methods

Descriptive statistics and outcome measures were assessed for normality, and statistical significance was assessed using independent samples *t*-test or Mann-Whitney *U* test. Based on their distribution, these are presented as mean (± sd) or median (first-third IQR). When HADS values were missing, the half-rule for imputation was applied (17). For the IES-R, the individual mean score was imputed for missing values when at least 75% of items were completed. Missing data in the outcomes analyzed were handled by the multilevel (mixed) models under the missing at random assumption. If missingness in covariates of the model was non-negligible, imputations were performed. All participants were analyzed according to their assigned treatment (“intention to treat” principle).

Continuous outcome variables were compared using linear multilevel models (including fixed effects for intervention and the different periods and a random cluster effect for patients nested within centers). In case of sufficient center periods with sufficient patients, models with different autocorrelation of centers over time were considered. Skewed distributed variables were log-transformed (when the minimum value was 0, the value +1 was log-transformed). Effects of such log-transformed variables were reported as the median ratio in the intervention versus the control condition on the original scale. Model fit was assessed using residual versus predicted plots, both at patient and center level, and by comparing the observed and predicted profiles of the centers over time. For the repeated surveys among HCPs, binary variables were compared similarly, using logistic multilevel models for which odds ratios (ORs) were reported. For all primary outcomes, their effect sizes were determined using Cohen’s d_s_ procedure.

All primary outcomes were statistically analyzed by an independent statistician (S.T.). Data were analyzed using IBM SPSS Statistics 27 (IBM Corp., Armonk, NY) and SAS 9.2 (SAS Institute, Cary, NC). Statistical significance was defined as *p* less than 0.05.

## RESULTS

**Figure 2.**
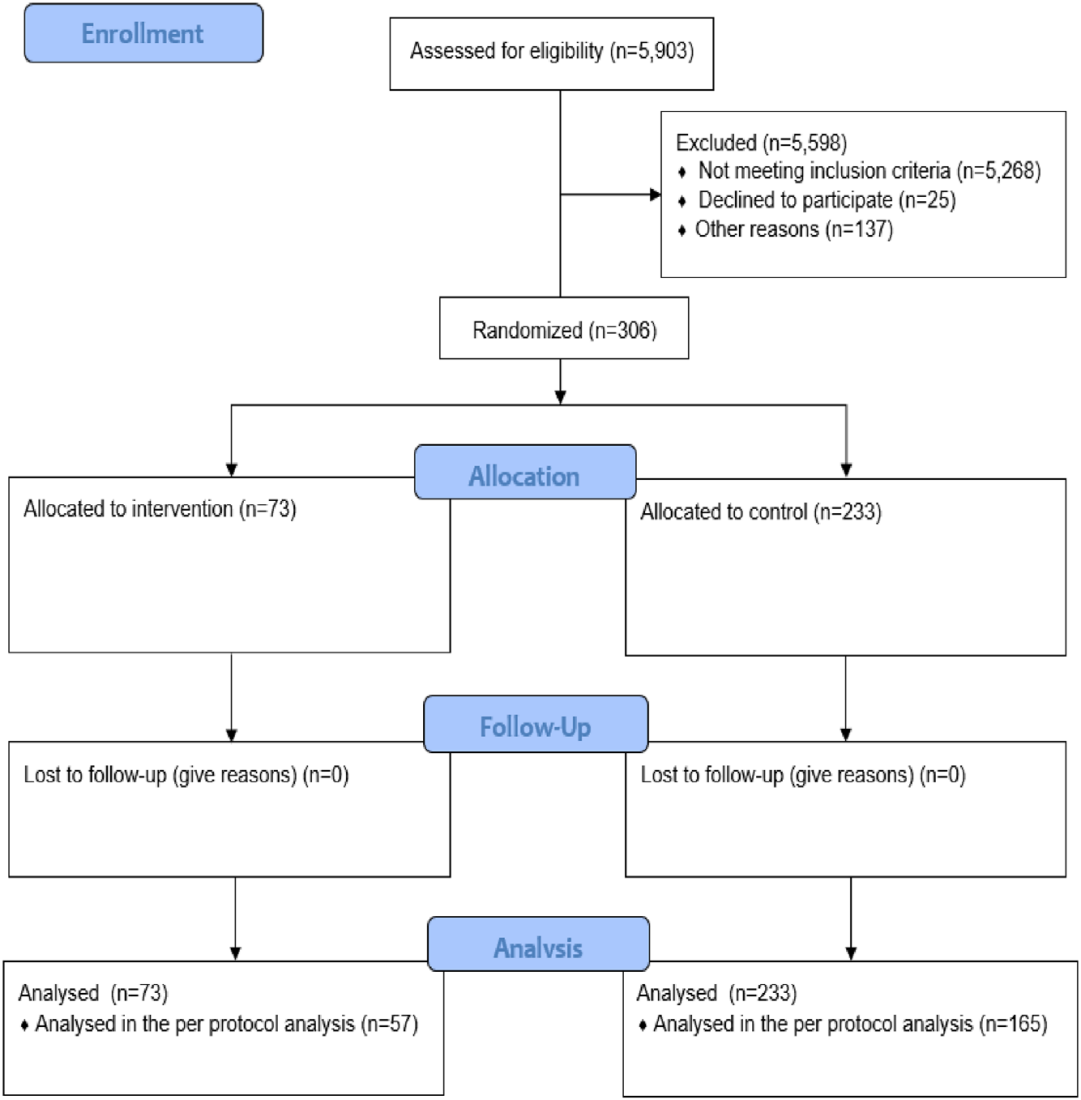
Flow diagram.

In the inclusion period, a total of 5.903 patients were admitted, of which 306 were included, 73 during the intervention period, and 233 during the control period (**Fig. 2**). Patients had a mean age of 62 (sd 15) years, had a median Clinical Frailty Score of 2 (IQR 2–4), and a median length of ICU stay of 10 (IQR 5–20) days. Relatives were mainly spouses (60%), had a mean age of 56 (sd 14) years, were mostly employed (*n* = 174, 59%) or retired (*n* = 94, 31%), and reported a good health status for themselves (**Table 1**).

**TABLE 1.** Demographics of Patients and Relatives

Demographics	Intervention (*n* = 73)	Control (*n* = 233)	*p*
Patients			
Age (yr), mean (sd)	63 (14)	62 (15)	0.46
Male, female	39 (54), 34 (46)	126 (54), 107 (46)	0.92
Admission type			
Medical	45 (62)	101 (43)	0.02
Surgical	17 (23)	43 (19)	
Neurology	8 (11)	56 (24)	
Trauma	3 (4)	32 (14)	
Clinical Frailty Score	3 (2–4)	2 (2–4)	0.54
Urgent admission	63 (89)	202 (87)	0.77
ICU length of stay	14 (8–26)	9 (5–17)	< 0.01
Mechanically ventilated	61 (84)	178 (78)	0.20
Days ventilated	9 (3–19)	5 (1–12)	< 0.01
Relatives			
Relation to the patient			
Partner, spouse	44 (61)	137 (60)	0.57
Parent	7 (10)	18 (8)	
Child	18 (25)	52 (22)	
Sibling	1 (1)	12 (5)	
Other	2 (3)	11 (5)	
Age (yr), mean (sd)	57 (14)	56 (14)	0.50
Male, female	29 (40), 43 (60)	82 (36), 148 (64)	0.48
Health status			
Excellent	15 (21)	48 (21)	0.15
Very good	28 (39)	60 (26)	
Good	25 (35)	96 (42)	
Moderate	4 (6)	21 (9)	
Bad	0 (0)	2 (1)	
Highest education level			
No formal level	1 (1)	3 (1)	0.72
Primary level	0 (0)	5 (2)	
Secondary level	48 (67)	138 (62)	
Higher level	23 (32)	71 (31)	
Other	0 (0)	11 (4)	
Current situation			
Scholar/student	1 (2)	1 (0)	0.41
Employment	38 (53)	97 (42)	
Self-employed	3 (4)	36 (16)	
Unemployed	0 (0)	4 (2)	
Unable to work	3 (4)	11 (5)	
Retired	24 (33)	70 (31)	
Other	3 (4)	10 (4)	
Hours of contract	31 (24–40)	32 (24–40)	0.58
Received support in completing survey	9 (14)	19 (9)	0.22

Data presented as *n* (%) or median (interquartile range) unless mentioned otherwise.

Regarding the implementation process, in all centers, the local working group was formed consisting of median of 6 (range, 4–8) members. A median of 3 (range, 2–4) training sessions per center were given, mostly live, but for two centers only online for COVID-19 pandemic reasons. The training was complemented by an online available digital version throughout the intervention period. The primary investigators (B.D., P.R., L.V.) contacted working group members every 3 months, by phone, video call, or newsletters, to discuss the study’s progress. The transition to the intervention period in the first three randomized centers was completed as planned. Because of the COVID-19 pandemic, one center was trained but decided to withdraw from transition to the intervention period, and implementation was delayed by 2 months in the last three randomized centers. Based on a limited number of included relatives and patients, related to increasing numbers of COVID-19 patients, the inclusion of participants was extended from 10 to 17 months, whereupon the inclusion was finalized for budgetary and logistical reasons. During the intervention period, relatives reported participating in median of 9 (IQR 2–17) of 34 possible essential care activities after implementation of the program. The most prevalent activities were pillow repositioning (*n* = 32, 60%), lip care (*n* = 31, 56%), and applying body lotion on hands (*n* = 30, 50%) (Supplemental file 3, http://links.lww.com/CCM/H442). No incidents or adverse events related to family participation were reported during the study.

### Primary Outcomes

For symptoms of anxiety, relatives in the intervention period reported a median HADS-A score of 5 (IQR 2–10) compared with 6 (IQR 3–9] in the control period (MR 0.72; 95% CI, 0.46–1.13; *p* = 0.15; d_s_ 0.02; 95% CI, –0.34 to 0.39). For symptoms of depression, a median HADS-D score of 4 (IQR 2–6) versus 3 (IQR 1–6) (MR 0.85; 95% CI, 0.55–1.32; *p* = 0.47; d_s_ –0.09; 95% CI, –0.46 to 0.28) was reported. For symptoms of posttraumatic stress, a median IES-R-score of 0.45 (IQR 0.27–0.82) versus 0.41 (IQR 0.14–1) (MR 0.94; 95% CI, 0.78–1.14; *p* = 0.54; d_s_ 0.01; 95% CI, –0.36 to 0.38) was reported. Reported satisfaction levels were slightly lower in the intervention period (mean 8.90 [sd 1.10]), compared with the control period (mean 9.06 [sd 1.10]; difference –0.60; 95% CI, –1.07 to –0.12; *p* = 0.01; d_s_ 0.15; 95% CI, –0.12 to 0.41) (**Table 2**; the per protocol analyses showed similar results [**Supplemental file 4**, http://links.lww.com/CCM/H442]). More relatives reported to have ever participated, 84.1% in the intervention period versus 74.7% in the control period (OR 3.43; 95% CI, 2.608–4.521; *p* < 0.001). In addition, 91% of the relatives in the intervention period appreciated participating in essential care (**Supplemental file 5**, http://links.lww.com/CCM/H442).

**TABLE 2.** Outcomes (Intention to Treat)

Outcomes	Descriptives		Estimates		
	Intervention	Control	Estimate (95% CI)	*p*	Intracluster Correlation Coefficient (95% CI)
Anxiety^a^	5 (2–10)	6 (3–9)	0.72 (0.46 to 1.13)^d^	0.15	0 (0 to 0.17)
Depression^a^	4 (2–6)	3 (1–6)	0.85 (0.55 to 1.32)^d^	0.47	0 (0 to 0.17)^f^
Posttraumatic stress disorder^b^	0.45 (0.27–0.82)	0.41 (0.14–1)	0.94 (0.78 to 1.14)^d^	0.54	0 (0 to 0.17)^f^
Satisfaction^c^	8.90 (1.10)	9.06 (1.10)	–0.60 (–1.07 to –0.12)^e^	0.01	0.02 (0.00 to 0.20)

a 0–21; 8 or higher indicates symptoms of anxiety/depression.

b 0–4, a mean score of 1.6 or higher indicates symptoms of posttraumatic stress disorder.

c 0: very unsatisfied—10: very satisfied.

d Median ratio.

e Difference.

f Natural log-scale.

Descriptives are presented as median (interquartile range) or mean (sd). Estimates are corrected for length of stay.

### Secondary Outcomes

A total of 778 HCPs were invited for the survey and 235 (30%) responded (**Supplemental file 6**, http://links.lww.com/CCM/H442; demographics and experiences of ICU HCPs). They reported that 90% of their team had a neutral to positive attitude toward family participation in essential care in the intervention period, compared with 75% in the control period (OR 3.09; 95% CI, 1.31–7.31; *p* = 0.01). According to HCPs, relatives more often knew how to participate (47% vs 22%; OR 3.15; 95% CI, 1.64–6.05; *p* < 0.01). Also, according to HCPs both patients (71% vs 52%; OR 2.21; 95% CI, 1.12–4.35; *p* = 0.02) and relatives (76% vs 54%; OR 2.73; 95% CI, 1.34–5.58; *p* = 0.01) were satisfied more often in the intervention period. Furthermore, HCPs reported that more relatives had sufficient knowledge (41% vs 16%; OR 3.56; 95% CI, 1.75–7.25; *p* < 0.01) and skills (44% vs 25%; OR 2.38; 95% CI; 1.22–4.63; *p* = 0.01) to participate. Also, more HCPs reported that they had enough time to apply family participation (54% vs 30%; OR 2.73; 95% CI, 1.40–5.32; *p* < 0.01) (**Table 3**).

**TABLE 3.** Survey Results: Proportions of ICU Healthcare Providers That Agreed to Questions, or Had a Neutral to Positive Attitude Toward the Statements

Survey Questions/Statements	Intervention (%)	Control (%)	OR (95% CI)	*p*
Perceptions of ICU healthcare providers				
Are you currently neutral-positive toward family participation in essential care?	90	85	1.65 (0.68–4.02)	0.27
Is your team currently neutral-positive toward family participation in essential care?	90	75	3.09 (1.31–7.31)	**0.01**
Are relatives currently neutral-positive toward family participation in essential care?	93	95	0.65 (0.18–2.34)	0.51
Are patients currently neutral-positive toward family participation in essential care?	71	72	0.92 (0.45–1.88)	0.81
Did you find it difficult to invite relatives to participate in essential patient care?	28	45	0.48 (0.23–0.97)	**0.04**
Did the relative take the initiative to participate in the past period?	32	34	0.88 (0.44–1.75)	0.71
Did uncertainty about the wishes of the patient play a role in family participation in essential patient care (protection of privacy)?	37	33	1.22 (0.58–2.58)	0.60
Did uncertainty about relatives burden play a role in family participation in essential patient care?	48	46	1.08 (0.54–2.16)	0.84
Was the relative capable of learning to participate in essential patient care?	58	53	1.24 (0.62–2.46)	0.54
Were there any activities that you did not consider safe for relatives to participate in?	65	63	1.09 (0.53–2.24)	0.82
Changes in care				
Family participation in essential patient care is easy to apply	60	47	1.66 (0.89–3.10)	0.11
It is clear to relatives how they can participate in essential patient care	47	22	3.15 (1.64–6.05)	**< 0.01**
Relatives are satisfied with participation in essential patient care	76	54	2.73 (1.34–5.58)	**0.01**
Patients are satisfied with the participation in essential care of their relative	71	52	2.21 (1.12–4.35)	**0.02**
I am satisfied with the participation in the essential patient care of the relative	52	47	1.22 (0.65–2.27)	0.54
Family participation in essential patient care takes a lot of effort	56	56	1.03 (0.55–1.95)	0.92
I have sufficient knowledge to apply family participation in essential patient care	84	67	2.48 (1.11–5.55)	**0.03**
I have sufficient skills to apply family participation in essential patient care	87	73	2.60 (1.07–6.30)	**0.04**
The relatives have sufficient knowledge to apply family participation in essential patient care	41	16	3.56 (1.75–7.25)	**< 0.01**
The relatives have sufficient skills to apply family participation in essential patient care	44	25	2.38 (1.22–4.63)	**0.01**
My job satisfaction increases by applying family participation in essential care	44	45	0.98 (0.51–1.85)	0.94
The application of family participation in essential patient care is a major change in my daily work	34	57	0.39 (0.20–0.76)	**0.01**
Contextual factors				
There is sufficient space in the ICU to apply family participation in essential patient care	55	42	1.69 (0.88–3.22)	0.11
There is sufficient privacy in the ICU to apply family participation in essential patient care	77	66	1.80 (0.86–3.79)	0.12
Visiting hours facilitate the application of family participation in essential patient care	46	41	1.22 (0.64–2.34)	0.55
I have enough time to apply family participation in essential care	54	30	2.73 (1.40–5.32)	**< 0.01**
I feel supported by the members of the project team to apply family participation in essential patient care	62	52	1.47 (0.76–2.84)	0.26
I feel supported by my manager to apply family participation in essential patient care	60	52	1.35 (0.70–2.61)	0.37
The application of family participation in essential patient care fits within the values of quality of care for patients in the ICU	90	74	3.38 (1.23–9.32)	**0.02**

OR = odds ratio.

Boldface font indicates *p* < 0.05.

Patient’s median length of stay in the ICU was 14 (IQR 8–26) days in the intervention period versus 9 (IQR 5–17) days in the control period (*p* < 0.01). Patient’s median days on ventilation was 9 (IQR 3–19) days versus 5 (IQR 1–12) days (*p* < 0.01) (Table 1).

## DISCUSSION

This study investigated the effect of a standardized program to facilitate family participation in essential care activities in daily ICU practice on PICS-F symptoms and satisfaction among relatives. Reported levels of anxiety, depression, posttraumatic stress symptoms, and satisfaction remained similar after implementation of the intervention. Furthermore, HCPs were mostly positive about the program and recognized increased clarity, knowledge and skills among relatives, and HCPs.

Our study was conducted based on recommendations in guidelines for family-centered care (FCC) in the ICU, to implement policies to promote FCC to improve relatives’ experience (4). Our findings align with a recent review, that found that proactive communication and provision of information did not lead to a reduction in prevalence of depression, anxiety, and PTSD in relatives (18), though our intervention was directed at active family participation instead of passive family involvement. Our findings differ from recent original studies on mental health symptoms, which may be explained by two important factors. First, the timing of measurements: based on the known expression of PICS-F symptoms, which may occur after 3 months postevent (2), we measured symptoms of anxiety, depression, and posttraumatic stress 3 months after ICU discharge. This differed from other studies that measured “acute anxiety levels” during the patient’s stay in the ICU (19–21). Second, compared with a study that measured PICS-F symptoms in a similar way (19) the reported baseline levels of anxiety, depression, and posttraumatic stress were already relatively low in our control period, and similar to levels in the intervention period in other contexts. For satisfaction, we showed a statistically significant but clinically irrelevant decrease. Important to realize, is that satisfaction scores were excellent in both periods and were similar to findings in similar studies during the intervention period (19, 21). Possibly, a “ceiling effect” may have been achieved. According to HCPs, both patients and relatives seemed significantly more satisfied with family participation in the intervention period, corresponding with most relatives having appreciated participating in essential care activities.

Third, the COVID-19 pandemic affected the conduct of the study, requiring a slight delay and minor adjustments in the implementation process, and extension of the inclusion period. The COVID-19 pandemic has affected relatives (22, 23), limiting possibilities for relatives to visit the patient and participate in essential care activities. Also, this may have caused differences in case mix, length of stay, length of mechanical ventilation, and outcomes that were determined. Differences in length of stay were corroborated in several studies (24, 25). However, based on similar studies that were previously described (18, 19, 21), our results seem robust. The COVID-19 pandemic has affected HCPs as well (26–29) and may have influenced intervention delivery, warranting further research.

## STRENGTHS AND LIMITATIONS

Our study had several strengths: it was a relatively large study with seven participating ICUs and a large number of participants. The program for family participation further facilitated relatives to help the patient, a need that has been expressed in several studies (3, 30–33). Furthermore, it enabled ICU nurses to further develop their role in supporting relatives and patients through family participation and enhance their leadership skills.

Some limitations have to be addressed. Outcome measures other than PICS-F symptoms might have reflected effects of family participation in essential care activities better. Previous work described, for example, experienced interest, comfort, respect, collaboration, and support (34–36). Furthermore, measurement of mental health symptoms among relatives only after 3 months may not have been sufficient, as patients’ rehabilitation trajectories vary. Similar studies collected longitudinal data before admission, as the mental health status of relatives before ICU admission may be an important predictor of mental health outcomes of relatives after the patient’s ICU stay (37) and following admission (18, 38, 39). Furthermore, as shown in this Dutch study, facilitating the process of participation was shown to increase the odds of a neutral to positive attitude toward participation, which probably will facilitate participation in daily ICU practice. This improved attitude calls for further research in other countries.

## CONCLUSIONS

Implementation of a standardized program for family participation in essential patient care in the ICU did not lead to a relevant change in satisfaction levels of relatives, nor in symptoms of anxiety, depression, or posttraumatic stress in relatives after 3 months. HCPs reported that several outcome measures, such as patient and relative satisfaction levels, as well as the clarity, knowledge, and skills of relatives and HCPs regarding family participation, increased after implementation of the program.

## ACKNOWLEDGMENTS

The authors thank the nursing and medical staff for their efforts, in particular the EFFAMPART working group members, as well as the relatives and patients for their cooperation during the study.

## Supplementary Material
