# Supplementary material for: Effect of a Standardized Family Participation Program in the ICU: A Multicenter Stepped-Wedge Cluster Randomized Controlled Trial*
Source: Crit Care Med. 2023 Nov 7;52(3):420–31. doi: 10.1097/CCM.0000000000006093 (PMC10876177; doi:10.1097/CCM.0000000000006093)
Supplement: Supplementary file 1 [file ccm-52-0420-s001.docx]

**Content supplemental files**

**Supplemental file 1: The EFFAMPART program menu P2**

**Supplemental file 2: Information posters EFFAMPART program P3-7**

**Supplemental file 3: What activities of the family participation program did relatives**

**perform? P8**

**Supplemental file 4: Outcomes (per protocol) P9**

**Supplemental file 5: Experiences of relatives P10**

**Supplemental file 6: Demographics and experiences of ICU healthcare providers P11**

**Supplemental file 7: Working group members P12**

**Supplemental file 1: The EFFAMPART program menu**

The menu below was printed double-sided, and laminated, and adapted for relatives with low-literacy or from different cultural backgrounds, who have difficulty reading Dutch. It contains similar information with pictures on the front, and text on the back.


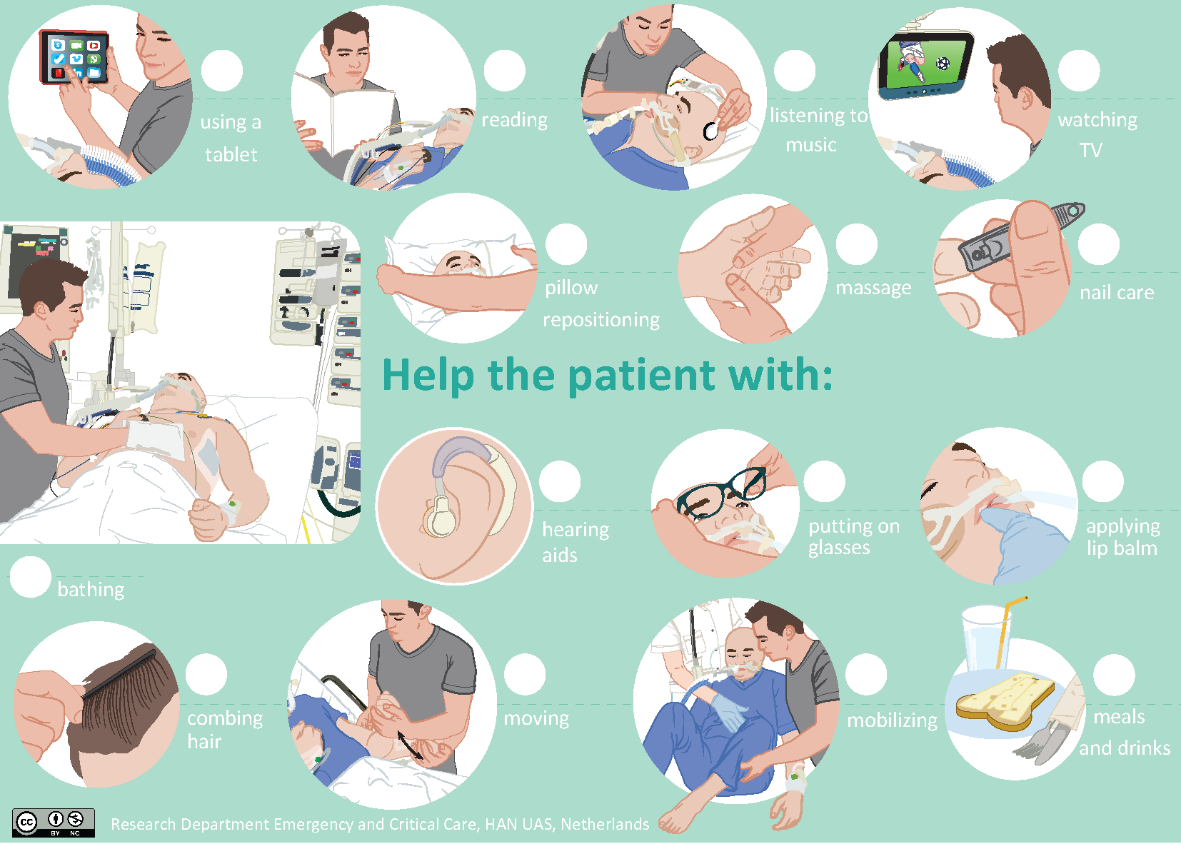


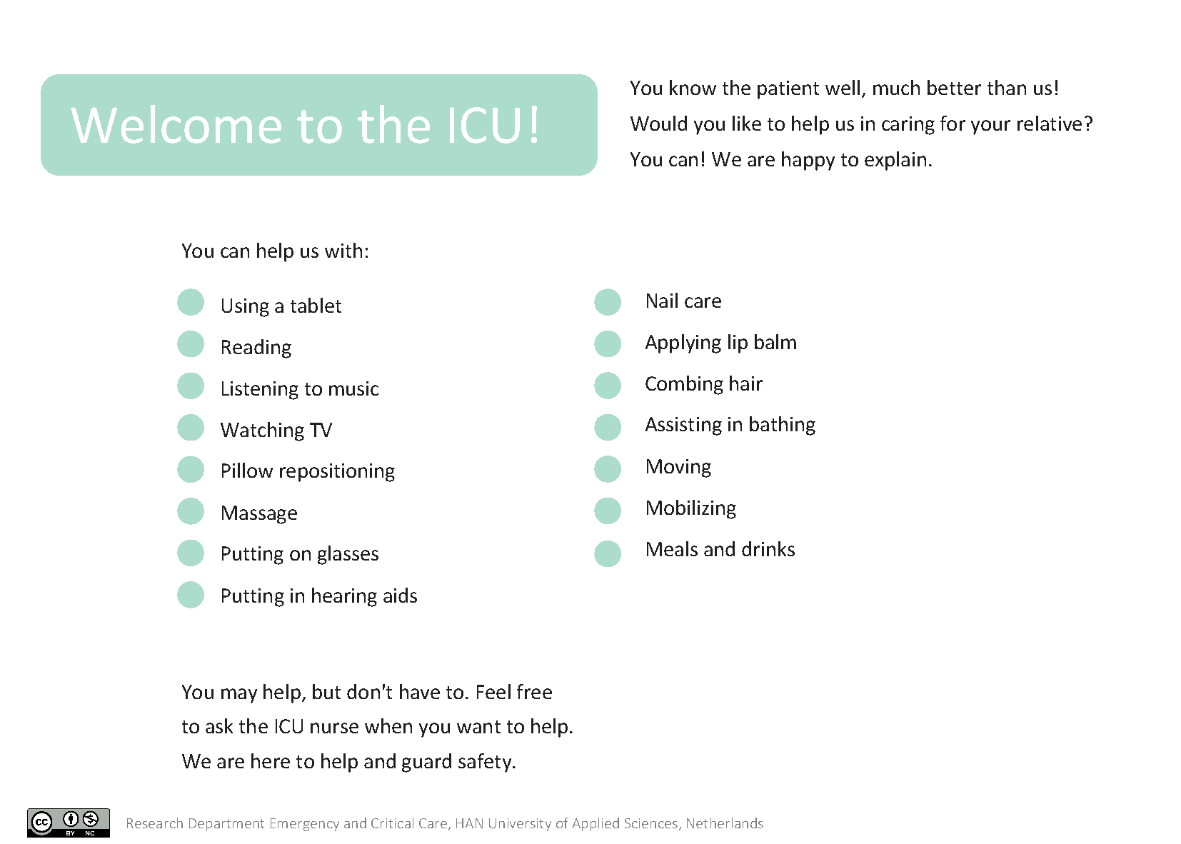


**Supplemental file 2: Information posters EFFAMPART program**


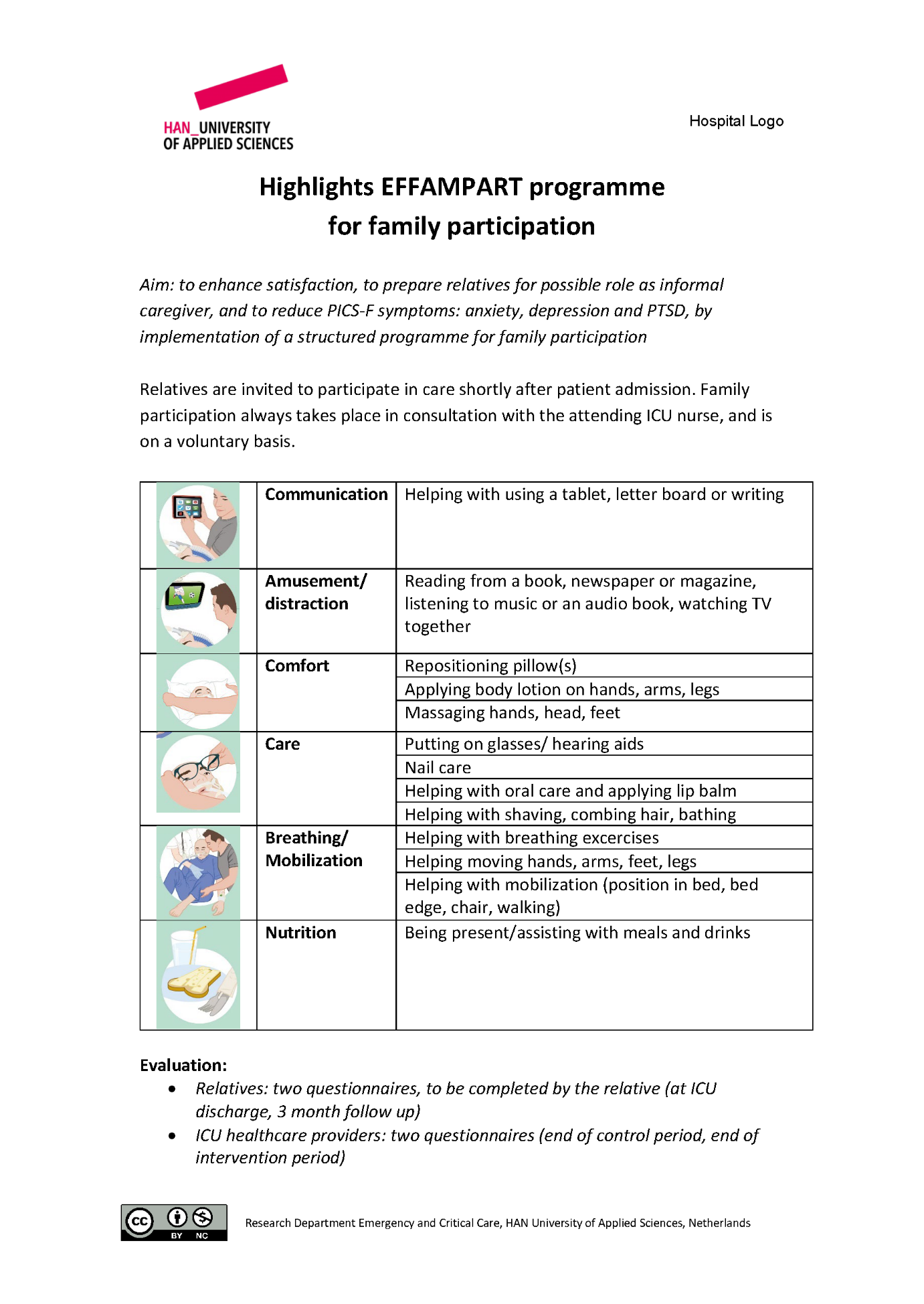


**Supplemental file 2: Information posters EFFAMPART program**


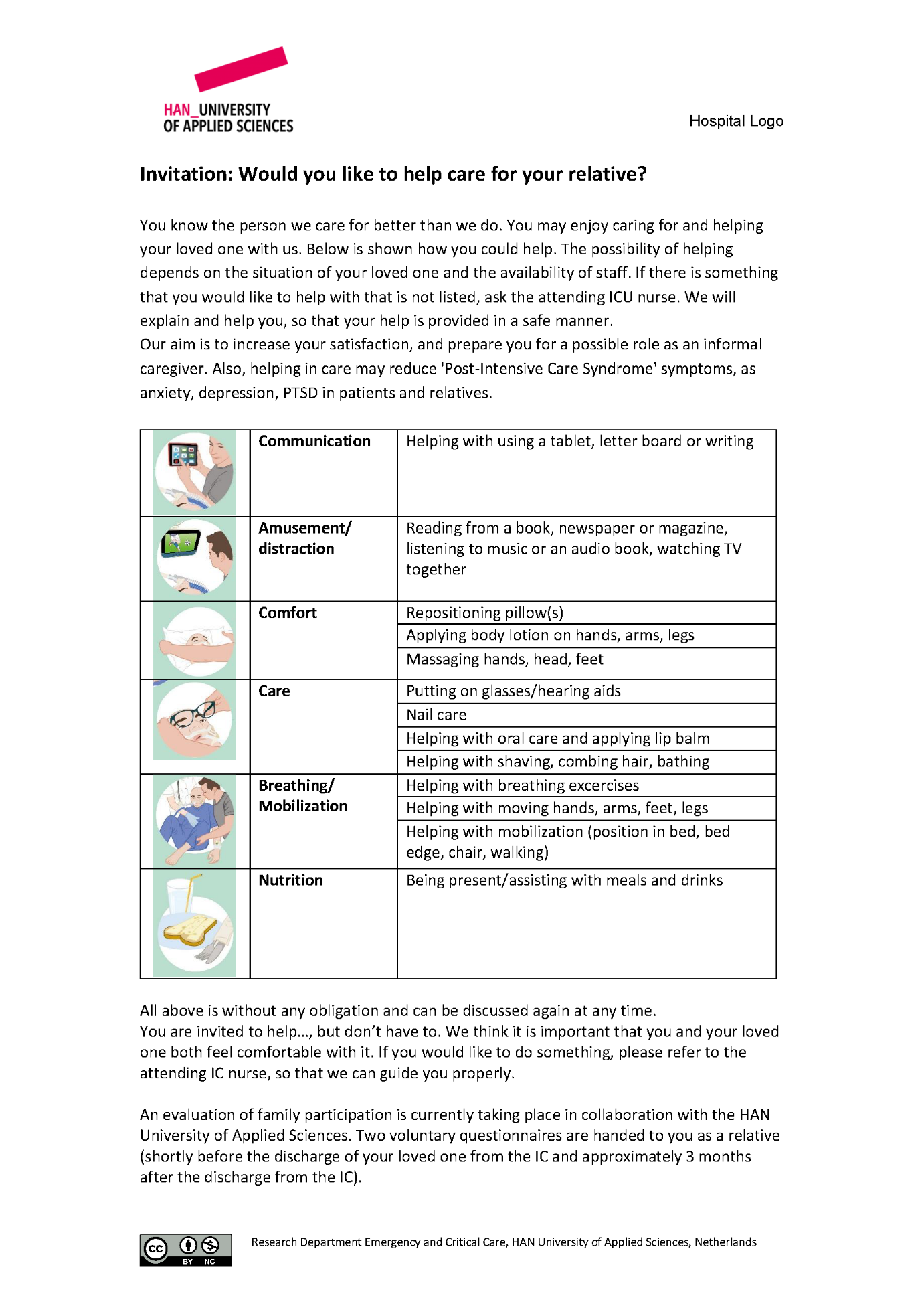


**Supplemental file 2: Information posters EFFAMPART program**

**Work instruction: Family participation in essential care activities in ICU patients.**

*Goal*

Enhancing satisfaction, preparing relatives for a possible role as informal caregiver and possibly reducing PICS(-F) symptoms: anxiety, depression, PTSD in patients and relatives; providing a clear and uniform working method for ICU healthcare providers.

*Terms, definitions and abbreviations*

PICS(-F): post-intensive care syndrome(-family): anxiety, depression, PTSD

PTSD: Post-Traumatic Stress Disorder

*Scope*

Patients admitted to the Intensive Care Unit of the hospital, and their relatives.

*Background, theory and principle*

This instruction contains a program, with possible essential activities for ICU patients, that relatives could participate in, that has been implemented in response to the EFFAMPART study.

*Responsibilities*

The ICU nurse is independently authorized, based on his/her own clinical expertise, to establish and coordinate with relatives of the ICU patient, and when possible with the patient himself, in what activities relatives could participate.

*Safety and environment*

Safety of the patient, ICU healthcare provider(s) and relatives is paramount. Family participation is free of obligation for relatives: relatives can participate if they wish, it is not obliged, it is important that the patient and relatives feel comfortable with it; family participation is a process: wishes and needs may change. Family participation depends on the physical and mental capacities of relatives. With family participation, the patient's situation (stability, phase of stay/length of stay) is taken into account. Relatives always participate in consultation with the attending ICU nurse and/or in the presence of the ICU nurse/attending healthcare provider.

*Method*

Relatives are informed about the possibility to participate in care activities, through a letter, poster (in family room), menu with possible activities and/or verbally, one/a few day(s) after admission. Relatives are presented with a menu with possible activities they can participate in, both in text and pictures (for relatives with low-literacy or from different cultural backgrounds, who have difficulty reading Dutch). Relatives receive the necessary instructions from the IC nurse/physical therapist/ speech therapist/other ICU healthcare provider on duty.

| 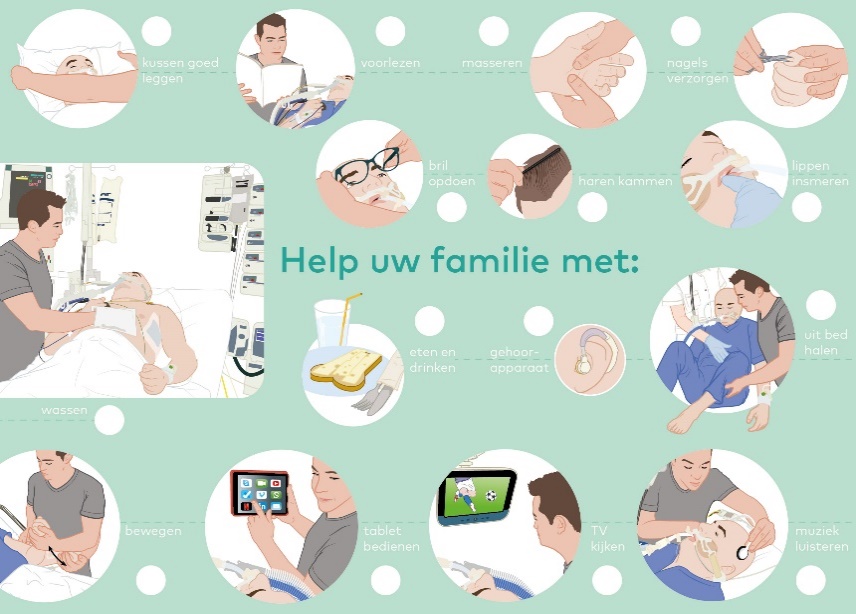 | **Communication** | Helping with using a tablet, letter board or writing |
| --- | --- | --- |
| 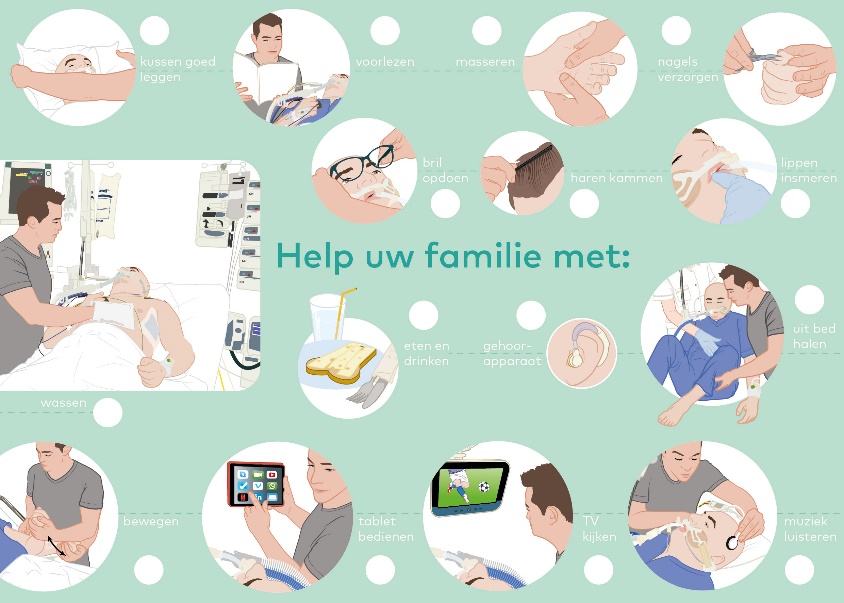 | **Amusement/**  **distraction** | Reading from a book, newspaper or magazine, listening to music or an audio book, watching TV together |
| 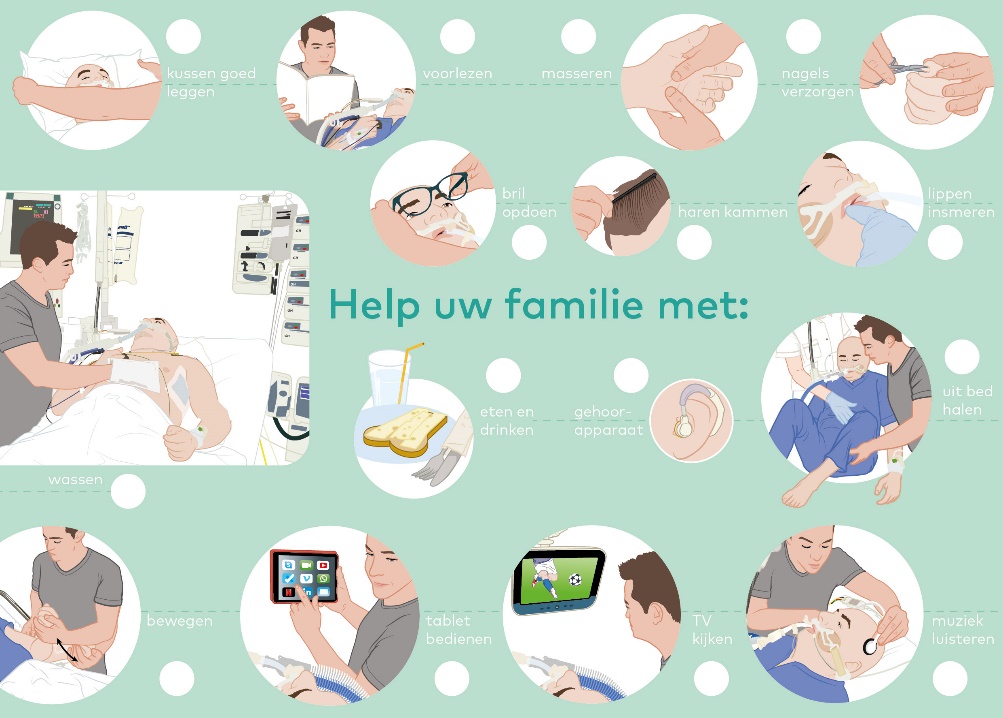 | **Comfort** | Repositioning pillow(s) |
|  |  | Applying body lotion on hands, arms, legs |
|  |  | Massaging hands, head, feet |
| 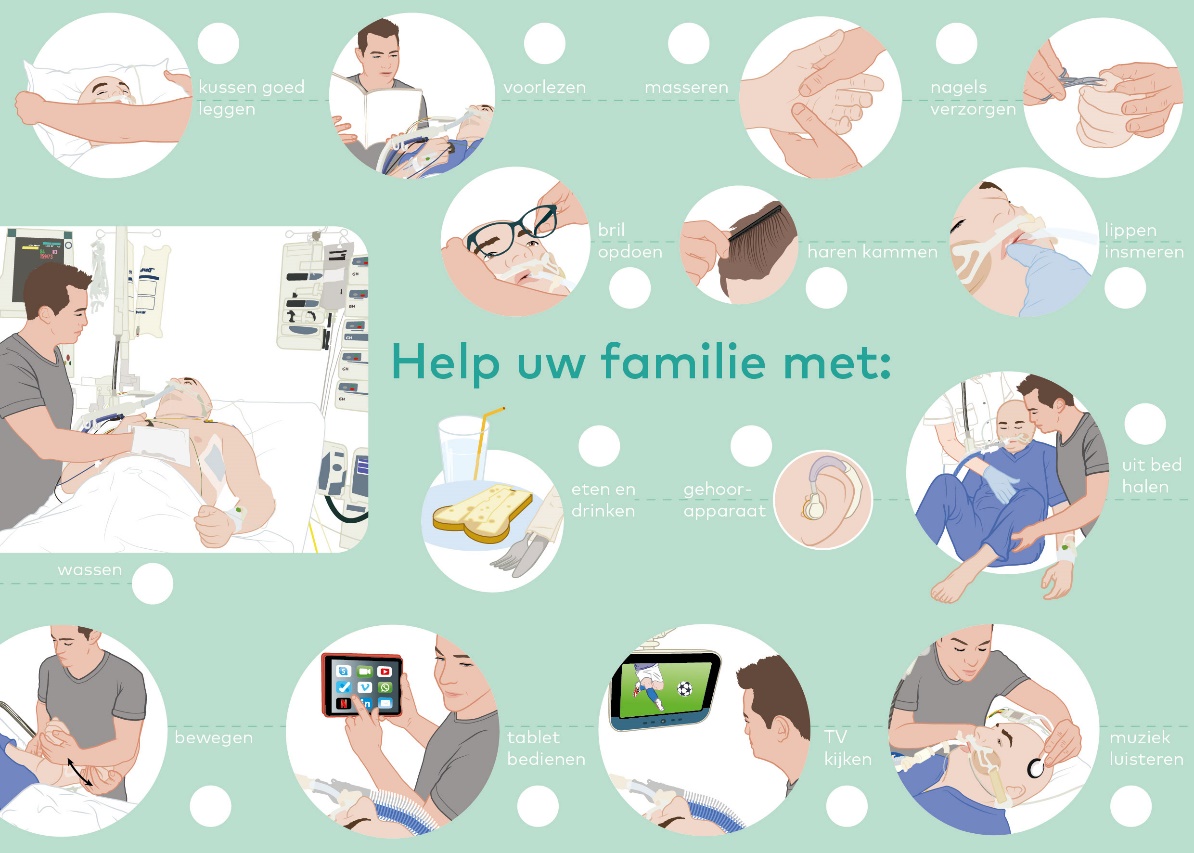 | **Care** | Putting on glasses/hearing aids |
|  |  | Nail care |
|  |  | Helping with oral care and applying lip balm |
|  |  | Helping with shaving, combing hair, bathing |
| 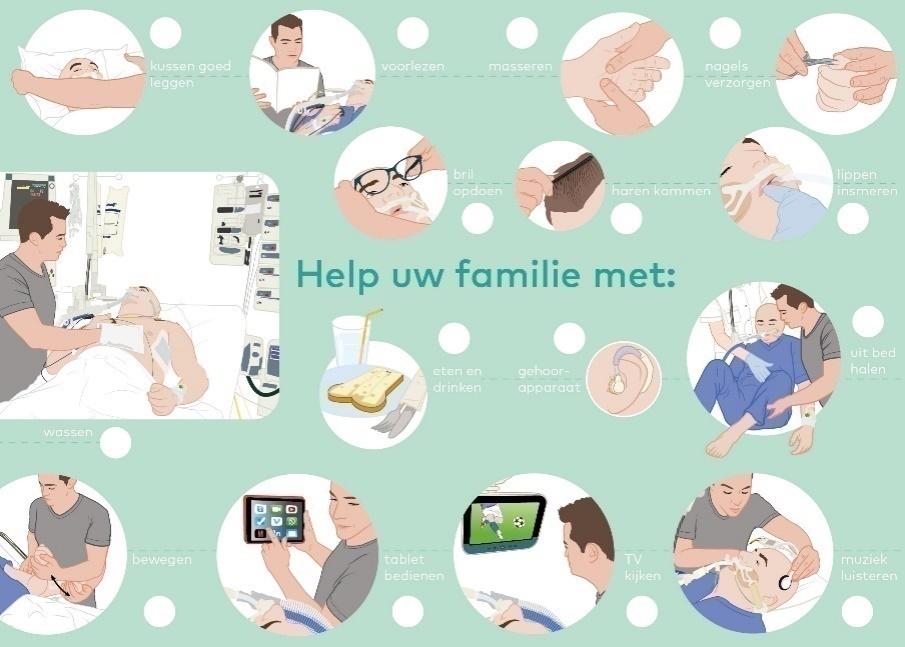 | **Breathing/**  **Mobilization** | Helping with breathing exercises |
|  |  | Helping with moving hands, arms, feet, legs |
|  |  | Helping with mobilization (position in bed, bed edge, chair, walking) |
| 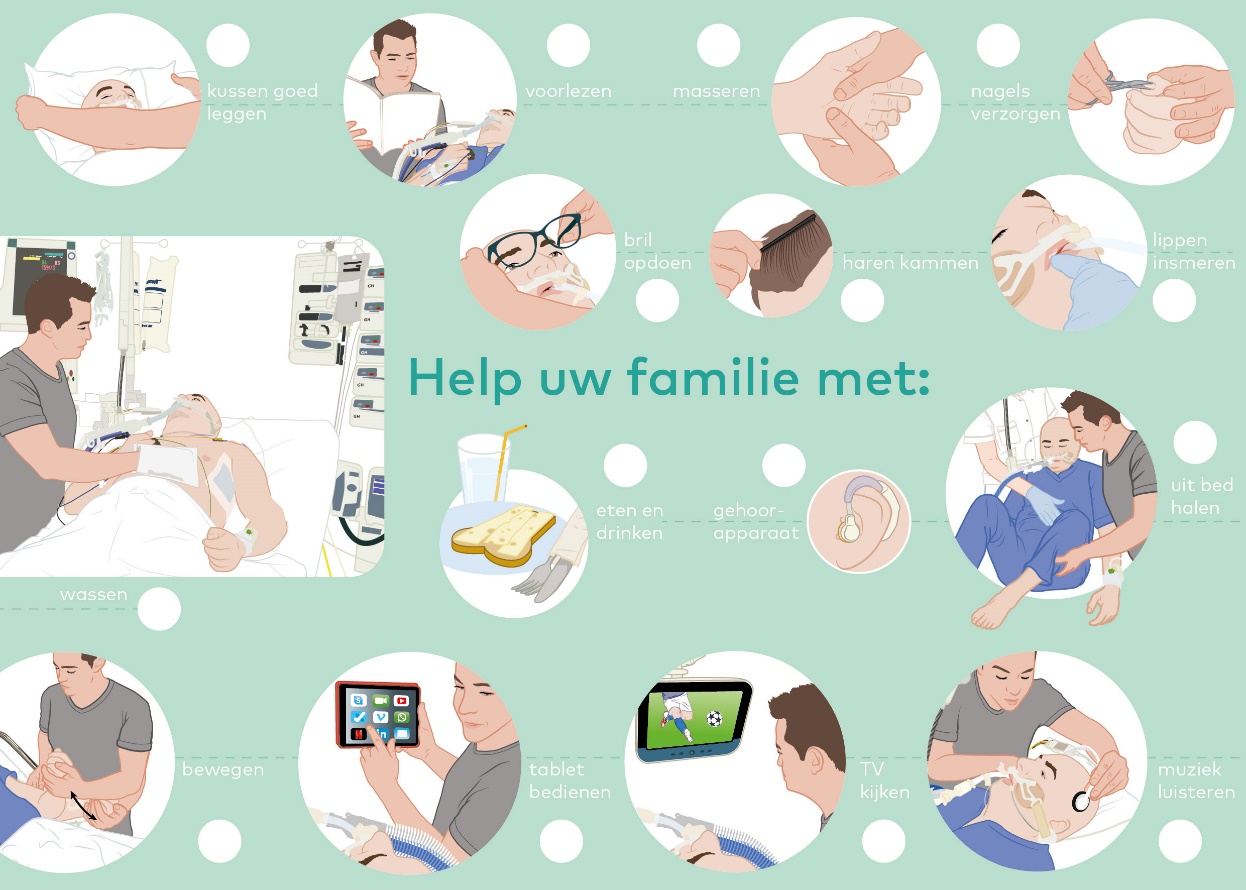 | **Nutrition** | Being present/assisting with meals and drinks |

*Reporting*

The ICU nurse reports agreements and results in the patient file.

*Points of attention*

Until October 2022, the effects of the activities will also be used for evaluation in the context of the EFAMPART study.

*References*

Azoulay et al; French Famirea Group. (2003) Family participation in care to the critically ill: opinions of families and staff. Intensive Care Medicine, 29(9): 1498-504.

Davidson et al. (2017) Guidelines for Family-Centered Care in the Neonatal, Pediatric, and Adult ICU. Critical Care Medicine, 45(1): 103-28.

Garrouste-Orgéas et al. (2010) Opinions of families, staff, and patients about family participation in care in intensive care units. Journal of Critical Care, 25(4): 634-40.

Hammond. (1995) Involving families in care within the intensive care environment: a descriptive survey. Intensive and Critical Care Nursing, (5): 256-64.

Heydari et al. (2020) Family participation in the care of older adult patients admitted to the intensive care unit: A scoping review. Geriatric Nursing 41(4):474-484.

Liput et al. (2016) A Review of the Perceptions of Healthcare Providers and Family Members Toward Family Involvement in Active Adult Patient Care in the ICU. Critical Care Medicine, 44(6): 1191-7.

McConnell, B. & Moroney, T. (2015) Involving relatives in ICU patient care: critical care nursing challenges. Journal of Clinical Nursing, 24(7-8): 991-8.

Mitchell et al. (2016) Patient, family-centred care interventions within the adult ICU setting: An integrative review. Australian Critical Care, 29: 179–193.

Olding et al. (2015) Patient and family involvement in adult critical and intensive care settings: a scoping review. Health Expectations, 19:1183–1202.

The Johns Hopkins Hospital (2010) Family Involvement Menu. http://www.hopkinsmedicine.org/armstrong_institute/_files/_documents/family_involvement_menu.pdf

**Supplemental file 3: What activities of the family participation program did relatives perform?**

| What activities of the family participation program did you perform? (n=54) | | |
| --- | --- | --- |
|  |  | **Yes (%)** |
| Communication | Helping with operating the tablet/ iPad | 37 |
|  | Helping with the use of the letter board | 26 |
|  | Helping with writing | 14 |
| Amusement/ distraction | Reading from a book, newspaper or magazine | 30 |
|  | Playing music or audio book | 42 |
|  | Watching favorite TV program together | 40 |
| Comfort | Repositioning the pillow(s) | 60 |
|  | Applying body lotion on hands | 50 |
|  | Applying body lotion on arms | 47 |
|  | Applying body lotion on legs | 48 |
|  | Giving a hand massage | 43 |
|  | Giving a head massage | 20 |
|  | Giving a feet massage | 40 |
| Care | Putting on glasses | 48 |
|  | Putting in hearing aids | 4 |
|  | Nail care | 39 |
|  | Helping taking care of lips | 56 |
|  | Helping with oral care | 34 |
|  | Helping with dental care | 29 |
|  | Helping with shaving | 20 |
|  | Helping combing hair | 37 |
|  | Helping washing hair | 14 |
|  | Helping with bathing | 12 |
| Breathing | Helping with breathing exercises | 15 |
| Mobilization | Helping with moving hands | 47 |
|  | Helping with moving arms | 40 |
|  | Helping with moving feet | 40 |
|  | Helping with moving legs | 35 |
|  | Helping with mobilization (changing position in bed) | 28 |
|  | Helping with mobilization (on the bed edge) | 28 |
|  | Helping with mobilization (in the chair) | 30 |
|  | Helping with mobilization (walking) | 18 |
|  | Helping with mobilization (in the swimming pool)* | 8 |
| Nutrition | Being present/assisting with meals and drinks | 48 |

***** Available in one setting only

**Supplemental file 4: Outcomes (per protocol)**

|  | *Descriptives* | | *Estimates* | | |
| --- | --- | --- | --- | --- | --- |
|  | **Intervention** | **Control** | ***Estimate (95%CI)*** | ***p*** | ***ICC (95%CI)*** |
| Anxiety^A^ | 2 [1-2] | 2 [1-2] | 0.74 (0.45 to 1.21)^D^ | 0.22 | 0 (0 to 0.20) |
| Depression^A^ | 2 [1-2] | 1 [1-2] | 0.87 (0.54 to 1.40)^D^ | 0.56 | 0 (0 to 0.20)^F^ |
| Post-traumatic stress disorder^B^ | 0.37 [0.24–0.60] | 0.36 [0.17-0.69] | 0.91 (0.71 to 1.16)^D^ | 0.41 | 0 (0 to 0.17)^F^ |
| Satisfaction^C^ | 8.89 (1.13) | 9.21 (0.96) | -0.62 (-1.09 to -0.15)^E^ | 0.01 | 0.02 (0.00 to 0.24) |

*Descriptives are presented as median [IQR] or mean (SD). Estimates are corrected for length-of-stay; ^A^0–21; 8 or higher indicates symptoms of anxiety/depression; ^B^0-4, a mean score of 1.6 or higher indicates symptoms of PTSD; ^C^0: very unsatisfied – 10: very satisfied; ^D^median ratio, ^E^difference; ^F^natural log-scale*

A per protocol analysis was conducted on relatives that indicated to have participated. The per protocol analyses showed similar results as analyses according to their assigned treatment (‘intention to treat’ principle).

**Supplemental file 5: Experiences of relatives**

| Opinions of relatives after implementation of the program for family participation (n=66) | |
| --- | --- |
|  | |
| Statement | Yes (%) |
| I would like to help in essential patient care for my significant other | 85 |
| I appreciated being able to do something in the care for my significant other | 91 |
| I felt free in my choice to help in essential patient care | 80 |
| My significant other appreciated that I could do something for him/ her in essential patient care | 92 |
| Did you feel invited by the ICU nurse to help in essential patient care? | 64 |
| Did you feel invited by other healthcare providers (nursing assistant / physical therapist / speech therapist) to help in essential patient care? | 50 |

| Experiences of relatives with family participation in daily practice (n=66) | |
| --- | --- |
|  | Yes (%) |
| It was clear to me how I could help in essential patient care | 62 |
| Helping in essential patient care took me a lot of effort | 20 |
| I had sufficient knowledge to help in essential patient care | 65 |
| I had sufficient skills to help in essential patient care | 68 |
| The ICU nurse asked if I wanted to do something | 50 |
| I indicated that I wanted to do an activity from the menu | 44 |
| I chose the activity from the menu myself | 54 |
| I was able to do the activity that I wanted | 69 |
| The ICU nurse encouraged me to help in essential patient care for my significant other | 42 |
| I found it scary to help in essential patient care for my significant other | 20 |
| I appreciated helping in essential patient care for my significant other | 87 |
| I felt obliged to help in essential patient care for my significant other | 7 |
| I felt that I could choose whether I wanted to help | 75 |
| There was enough room in the ICU to help in essential patient care for my significant other | 72 |
| There was enough privacy in the ICU to help in essential patient care for my significant other | 88 |
| The visiting hours allowed me to help in essential care for my significant other | 60 |
| The ICU nurse had time to guide me through helping in essential patient care for my significant other | 63 |

**Supplemental file 6: Demographics and experiences of ICU healthcare providers**

A total of 778 ICU healthcare providers were invited, 235 (30%) responded. Of the responders, 201 (86%) were in the first round and 43 (18%) in the second round. Of the responders, 173 (74%) were female, 185 (79%) indicated to be an ICU nurse, and had a median of 12 years [IQR 5-25] of experience in the ICU.

| Demographics | n=235 |
| --- | --- |
| Gender (n, %) Female  Male  Unanswered | 173 (74)  28 (12)  34 (15) |
| Position (n, %) ICU nurse  ICU nurse trainee  Medium care nurse  Manager  Nurse specialist  Other  Unanswered | 185 (79)  5 (2)  4 (2)  2 (1)  1 (1)  3 (2)  35 (13) |
| Years of experience (median [IQR]) | 12 [5-25] |
| Highest level of education (n, %) Secondary vocational level  Higher professional level  Post higher professional level  Academic level  Other  Unanswered | 39 (16)  101 (43)  53 (23)  6 (3)  1 (1)  35 (15) |

**Supplemental file 7: Working group members**

The authors gratefully acknowledge the efforts of the EFFAMPART working group members, who facilitated the conduct of the study, as well as the implementation of the intervention.

*Department of Intensive Care Medicine, Bernhoven, Uden, the Netherlands*

Esther Ewalds, Anouk Rovers, Esther de Bruijn, Wendy Wittenberg, Ellen Geldof

*Department of Intensive Care Medicine, Elisabeth Tweesteden Hospital, Tilburg, the Netherlands*

Anne Rutten, Erica Bekers, Ingeborg Schoenmakers

*Department of Intensive Care Medicine, Isala, Zwolle, the Netherlands:*

Crista Leerentveld, Kim van Dartel-Dorgelo, Hadewey Kiekebos-Terdu, Jany Krist-Meppelink, Alice Pap-Brugmans*,* Johanneke Spanjer-Eilerts*,* Felicia Veen-Schra*,* Arne Wentink

*Department of Intensive Care Medicine, Máxima Medical Center, Veldhoven, the Netherlands:*

Toine Klarenbeek, Mariem Aghmiri, Danielle Castelijns-Bakker, Sandy Charmant, Henriëtte Ederveen-Van Tiggelen, Aart Osinski, Demi Westerlaan

*Department of Intensive Care Medicine, Radboud university medical center, Nijmegen, the Netherlands:*

Mark van den Boogaard, Tessa Brouwer, Nicky Eijkenboom-Wattel, Marissa Jaspers, Lisette Kremers-van Hees, Ed van Mackelenberg, Suzan Meijer-Wijting, Lianne Vrieselaar

*Department of Intensive Care Medicine, Rijnstate, Arnhem, the Netherlands:*

Dominique Burgers, Helene Vogelesang, Yvonne Teitink

*Department of Intensive Care Medicine, Hospital Gelderse Vallei, Ede, the Netherlands:*

Marianne Bouw, Lydia, Barbara Festen-Spanjer, Yvonne Swaen-Dekkers
